# Effects of a Mobile App Called Quittr, Which Utilizes Premium Currency and Games Features, on Improving Engagement With Smoking Cessation Intervention: Pilot Randomized Controlled Trial

**DOI:** 10.2196/23734

**Published:** 2020-12-14

**Authors:** Ivan Bindoff, Tristan R Ling, Peter Gee, Benjamin Geelan, Stuart G Ferguson, Gregory M Peterson

**Affiliations:** 1 School of Pharmacy and Pharmacology University of Tasmania Sandy Bay Australia; 2 Tasmanian School of Medicine University of Tasmania Hobart Australia

**Keywords:** smoking, cessation, Quittr, engagement, retention, churn, cigarette, mHealth, game

## Abstract

**Background:**

Numerous mobile health (mHealth) apps have been developed to support smokers attempting to quit smoking. Although these apps have been reported to be successful, only modest improvements in the quit rate have been measured. It has been proposed that efforts to improve user engagement and retention may improve the quit rate further. Owing to the high cost of smoking-related disease, it is considered worthwhile to pursue even small improvements.

**Objective:**

The aim of this study was to test a novel smartphone app that leverages premium currency strategies developed by the mobile games industry in an attempt to improve engagement and retention with a smoking cessation intervention.

**Methods:**

We designed and developed a smoking cessation app called “Quittr” in line with previously developed smoking cessation mHealth apps. In addition to this established framework, we added a stand-alone fully featured city-building clicker-style game called “Tappy Town,” and a premium virtual currency called “QuitCoins.” The user earns QuitCoins for using the app in a way that contributes positively toward their quit attempt, and they can redeem these coins in Tappy Town for bonuses. To establish whether these features improved engagement and retention, we ran a 5-month randomized controlled trial where the intervention group had the full app with the extra games features, while the control group had the standard app only. Recruitment was performed via web-based advertising. Participants (N=175) had no direct contact with the researchers or other support staff.

**Results:**

No significant differences in terms of engagement, retention, or smoking outcomes were found between the control and intervention groups. However, survey data indicated that the majority of the participants valued Tappy Town (10/17, 59%) and the QuitCoins rewards system (13/17, 77%). Usage data also suggested that Tappy Town was widely played and was generally appealing to users (mean total time spent in app, control group: 797 seconds vs intervention group: 3502 seconds, *P<*.001). Analysis of the results suggests that users in the intervention group may have been negatively affected by the aspects of the chosen design, and some theories were explored to explain this unexpected outcome.

**Conclusions:**

Although the novel features of the Quittr app failed to improve the key outcomes measured in this study, there were enough positive indications to warrant further exploration of the concept. Additional research will be required to identify and correct any design flaws that may have adversely affected our participants before a follow-up study can be completed.

**Trial Registration:**

Australian and New Zealand Clinical Trials Register ACTRN12617000491369; https://www.anzctr.org.au/Trial/Registration/TrialReview.aspx?id=372661&isReview=true

## Introduction

Cigarette smoking is a major preventable cause of death, disease, and financial burden. Quitting smoking is the best thing an individual can do to reduce their risk of developing smoking-related diseases [1]. Providing smoking-specific behavioral support (eg, education, advice, assistance with goal-setting) is known to improve the likelihood of succeeding in a quit attempt [2], but effectively delivering such support and keeping the individual engaged with the process for a sufficient duration is difficult.

Mobile health (mHealth) apps and web-based support tools have typically only produced smoking cessation outcomes in the order of ~9.5% success rates. Although this is a clear improvement over unassisted quit attempts and represents potentially significant savings in avoided disease burden, the outcome is still modest [3]. The difference between the known potential of this content and the actual outcomes achieved via mHealth apps may be attributed to lack of user engagement and retention [4].

This problem is similar to that faced by mobile game designers, when attempting to produce profitable mobile games that collect revenue from in-app purchases and advertising—the so called “free-to-play” games. Games are expensive to produce. However, in-game advertising typically only returns up to a few cents per ad impression. Therefore, it is critical not only to reach a very large audience but also to ensure that the audience is retained over a relatively long time span. Similarly, with in-app purchases, it is generally necessary to ensure that users are thoroughly engaged with the game experience before they are enticed with paid content that they would believe would further enhance their experience. Recognizing these similarities, we developed a smoking cessation mHealth app, which leveraged tactics from the mobile games industry to drive engagement and retention. Instead of using these tactics to drive ad impressions or in-app purchases, our objective was to use them to encourage user engagement with behavioral support content and stay on track with their quit attempt for a longer time span. We called this app Quittr. The design process and philosophy that we used has been described in our earlier paper [5].

Previous smoking cessation mHealth apps included gamification elements to encourage user engagement and provide a sense of achievement and progression, and many included mini-games to keep the smokers’ hands occupied and to distract them from cravings [4,6-9]. Quittr also includes these elements as standard. However, to our knowledge, Quittr is the first such app to trial the inclusion of a fully-featured standalone game and a premium currency reward system.

For this pilot study, our aim was to determine whether these novel features added to the Quittr app could improve engagement with the smoking cessation support content and retention of the user making the quit attempt. The secondary outcome was to determine whether there was any effect on smoking cessation.

## Methods

### App Design

The Quittr app was broadly based upon existing smoking cessation apps (particularly SmokeFree-28 [4]) and standard behavioral content from other support tools. We included the following base features: (1) recording of daily cigarette usage, (2) a survey tool for collecting data about the user, (3) a dashboard with visualized statistics about changes to the user’s health and financial position, as calculated based on user-provided data, (4) an achievement system, to reward using the app and smoke-free periods, (5) an information toolbox, with over 12,000 words of quit smoking information and advice, broken into categories/sections, and simple multiple choice quizzes at the end of each section, (6) a distraction mini-game called “Hidden Object Game,” and (7) a daily notification system to remind the user to check-in and report their cigarette usage and to remind them to complete the exit survey (when appropriate).

In addition to these core features, we also added a fully featured game called “Tappy Town.” This game was designed to be useful to distract the user from cigarette cravings as well as to provide a long-term incentive to engage with the broader Quittr app. We chose a simple city builder style game since it was considered both achievable within budget/time frame and to be broadly appealing to potential users across the gender and age spectrum. We also intentionally avoided any potential smoking triggers by avoiding the topic of smoking within the game.

The general design mantra throughout development was that the user could not do anything to sabotage or adversely affect their gameplay experience and could thus play without any stress. However, they could plan and optimize their play to progress faster, rewarding effort and hopefully helping to make the game more engaging.

The game was designed as a “clicker-style” game, wherein the player would gradually accrue resources and then spend these resources to build more structures, which would in turn help them to generate resources faster. As they progress, the player unlocks more expensive and better structures, and the economy generally follows an exponential growth curve. This game model has proven in recent years to be highly compelling, at least for a few days of play, as the player chases down bigger and bigger numbers and unlocks grander structures with a strong sense of progression [10].

**Figure 1:**
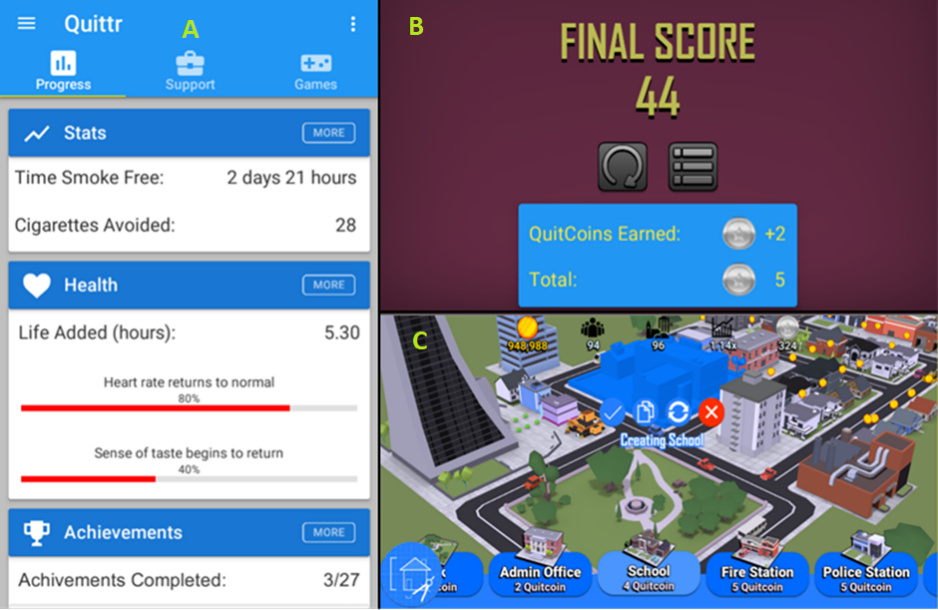
Examples of various screens in the Quittr app. A. Main dashboard; B. Earning QuitCoins after playing a distraction mini-game; C: Placing a new building in the Tappy Town game.

We made some modifications from the traditional model to help the smoker to quit. As well as the city passively earning currency, the game rewarded repeat interaction from the players’ hands by asking them to tap coins, which would periodically generate above their buildings to collect bonuses, as can be seen in Figure 1C.

We also tuned the game to encourage playing in short sessions at regular intervals throughout the day, which was roughly aligned with the frequency with which we would expect someone who is quitting smoking to experience cravings. Our intention here was that whenever someone was experiencing withdrawals, they could log in to the game and productively play a 2-5-minute session that would help them through the worst of their symptoms, without excessive interruption of their real life.

Additionally, users could earn QuitCoins, a type of premium currency [11] by using the broader Quittr app. They could then redeem these QuitCoins in the Tappy Town game to purchase powerful bonus structures, which were otherwise not possible to be purchased. Users were rewarded with QuitCoins via the achievements system when they (1) had a breakthrough smoke-free period, (2) avoided a certain number of cigarettes, (3) checked their statistics for the first time that day, (4) engaged with the information toolbox content for set periods of time, (5) successfully completed an information toolbox quiz, (6) played Tappy Town for the first time in a day, (7) played the Hidden Object Game for the first time in a day, or (8) set savings goals.

This mechanism was intended to facilitate the transfer of the player from the game back to the helpful quit smoking content and vice versa, and it was intended that the player would be motivated to engage with the quit-smoking content more closely than they might have otherwise, in order to advance in the Tappy Town game. However, little to no explicit messaging about these features was provided to the user. They were instead expected to experience this effect organically without any of the “nag screens” that would typically accompany this type of design in free-to-play mobile games. This was done to avoid adding potential stressors.

### Experimental Design

To test our innovation, we designed a randomized controlled trial with 2 groups: control and intervention (Multimedia Appendix 1). The intervention group had access to the full Quittr experience, including our games-based innovations and the QuitCoins premium currency incentivization system. The control group also had access to Quittr, but they did not have access to the Tappy Town game and could not earn QuitCoins. The protocol was registered with the Australian and New Zealand Clinical Trials Register [ACTRN12617000491369] and ethics approval was granted by the Tasmanian Health and Medical Human Research Ethics Committee [H0016506]. Participants were eligible if they were current smokers interested in starting a quit attempt, with a suitable smartphone device, aged 15 years and over, and spoke English. Allocation was done in a pseudo-random manner that was not known to participants, to ensure roughly equal group sizes. At semifrequent periods throughout the day, an algorithm was run to determine which group currently had more members and at which point the group with the fewest members would become favored for new registrations.

### Recruitment

Recruitment was open from December 12, 2017 through March 9, 2018 and was done primarily through posts on web-based forums and targeted advertising through Facebook (Multimedia Appendix 2). Participants were able to trial the app in testing mode and start a quit attempt at any time. Once they started a quit attempt, we tracked their data over a 28-day period. Although a few users elected to abandon 1 quit attempt and start a new quit attempt during the trial period, each quit attempt was evaluated individually, with the participant’s longest attempt being used for the final analysis.

### Data Collection

We logged all participant activity within the app, which was automatically uploaded to our server when internet access was detected via Wi-Fi. To answer the research questions on engagement and retention, we analyzed how frequently the participants were using the app, time spent using the various features of the app, and the time when usage occurred. We also administered basic surveys from within the app: an entry survey at the commencement of a quit attempt and an exit survey when the quit attempt was finished (28-day window completed) or abandoned (user-indicated) (Multimedia Appendix 3).

### Analysis

To determine whether the groups were equivalent in terms of participant characteristics at baseline, we used chi-square tests to compare categorical variables and two-sided independent samples *t* tests to compare means. When comparing the groups and outcomes at the end of the trial, we used means and two-sided independent samples *t* tests wherein data were parametric and medians and two-sided Wilcoxon rank sum tests wherein data were nonparametric. To explore engagement, we compared the time spent using the various features of the app between groups and analyzed retention by comparing the last day of usage between the groups. An equivalent test was also run to determine whether 1 group had a greater success at quitting smoking by comparing the longest smoke-free period of the members of each group. We used a chi-square test to determine differences in user perceptions indicated by the exit survey results and descriptive statistics to explore questions that were unique to 1 group only.

## Results

### Participant Characteristics

During the 4-month recruitment window, we recruited 182 participants. Unfortunately, an issue was found in the first week, which caused 6 participants to have their group incorrectly coded; thus, their results were discarded, leaving us with 176 participants and 209 quit attempts. An additional user was excluded for submitting unrealistic results, leaving us with 87 participants in the control group and 88 in the intervention group (N=175), although 23 did not submit survey responses beyond age and gender (12 in control and 11 in intervention). The 2 groups were well matched, as shown in Table 1.

**Table 1:** Comparisons of the demographic and smoking characteristics, based on entry survey data, between the control and intervention groups (N=175).

Demographic/smoking characteristics			Control group (n=87)		Intervention group (n=88)		*χ* ^2^ *(df)*		*P* value
Age (years), mean (SD)			39.5 (12.75)		40.5 (15.48)		N/A^a^		.36
**Gender, n (%)**							0.73		.24
	Female	44 (51)		44 (50)					
	Male	43 (49)		44 (50)					
**How soon after waking do you smoke your first cigarette? (minutes), n (%)^b^**							3.62 (4)		.46
	<5	24 (32)		24 (31)					
	6-15	18 (24)		20 (26)					
	16-30	17 (23)		11 (14)					
	31-60	11 (15)		11 (14)					
	>60	5 (7)		11 (14)					
How many cigarettes do you smoke per day on average? mean (SD)			20.27 (20.36)		19.53 (22.48)		N/A		.83
**Are you intending to use nicotine replacement therapy? n (%)^b^**							0.21 (1)		.65
	No	46 (61)		50 (65)					
	Yes	29 (39)		27 (35)					
**Are you intending to use medication to manage cravings/withdrawals? n (%)^b^**							0.20 (1)		.66
	No	67 (89)		67 (87)					
	Yes	8 (11)		10 (13)					

^a^N/A: not applicable.

^b^For these categories, only 75 participants in the control group and 77 participants in the intervention group provided responses.

### Participant Engagement With Quittr

Participants in the intervention group did post higher overall app usage statistics (mean time, intervention group: 3502 seconds vs control group: 797 seconds, *P<*.001) (Table 2). However, it appears that this extra time was almost entirely spent using the Tappy Town game. There were no other significant differences in the amount of time spent between the control and intervention groups, although the intervention group did trend toward spending additional time using the information toolbox (mean 189 seconds [intervention] versus 154 seconds [control]). The finance stats were close to statistical significance, but with only a little over 3 seconds separating them was not considered meaningful.

**Table 2:** Time spent by both the groups using the various features of Quittr.

Features of Quittr	Control group, average time, (seconds)	Intervention group, average time, (seconds)	Mean difference (seconds)	Standard error difference (seconds)	*P* value
Main dashboard	312.64	364.42	–51.78	74.50	.22
Information toolbox	154.39	188.79	–34.40	52.87	.11
Health stats	33.54	32.69	0.85	12.82	.84
Smoking stats	27.91	17.65	10.26	8.89	.19
Finance stats	6.96	10.65	–3.69	3.70	.06
Achievements	20.70	25.95	–5.25	9.79	.22
Tappy Town game	0.00	2651.78	–2651.78	709.97	N/A^a^
Hidden object game	132.81	99.89	32.92	32.79	.65
Total time in app	796.65	3501.86	–2705.21	816.73	*<.001* ^b^
Total time (excluding Tappy Town)	796.65	850.08	–53.43	167.43	.42

^a^N/A: not applicable.

^b^This result is significant at *P*<.05.

**Figure 2:**
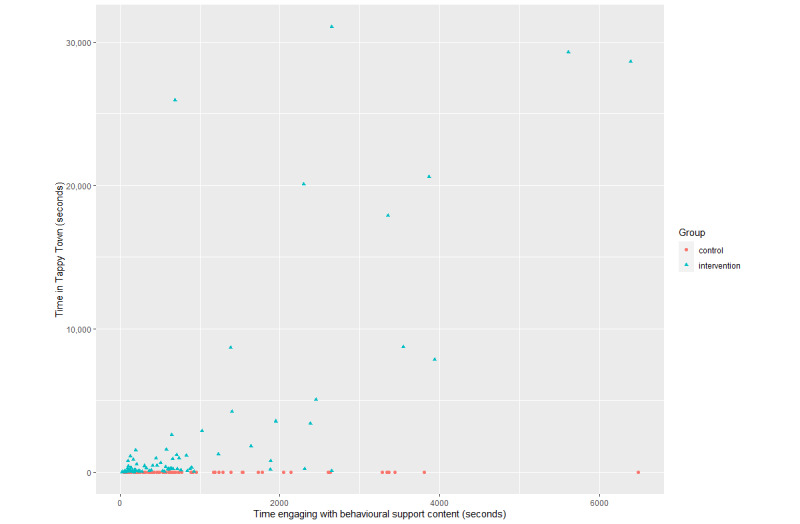
Time spent engaging with behavioral support content versus time spent in Tappy Town.

It was anticipated that the premium currency approach might be compelling only for a minority of users; therefore, the effect may not be demonstrable through comparison of the means. It is possible to visually inspect the outliers by plotting the time spent engaging with Tappy Town against the time spent engaging with the behavioral support content in the app (Figure 2).

### Participant Retention

The mean (SD) retention time of our participants was 6.67 (8.88) days. There was no statistically significant difference in the median number of days of app use between the control and intervention groups (median 2, IQR=0-12 vs median 1.5, IQR=0-9 days, respectively; *P=*.17).

### Smoking Status of the Participants

Only 7 of our 175 participants (4.0%) recorded 28 days smoke-free, with a mean (SD) smoke-free period of 4.80 (7.40) days. This result contrasts starkly with the results of the SmokeFree-28 app upon which Quittr was based, which saw 18.9% of their participants successfully achieve 28 days smoke-free. There was no statistically significant difference in the maximum achieved smoke-free period between the control and intervention groups (median 1, IQR=1-3.50 vs median 1, IQR=1-5 days, respectively; *P=*.59).

### Survey Responses

A total of 27 (31%) participants in the control group (n=87) submitted exit survey responses compared to only 17 (19%) for the intervention group (n=88) (*P=*.08). Although not reaching significance, this apparent disparity in the submissions was an unexpected outcome. An additional two-sided *t* test was performed to determine if the baseline smoking profile of the participants who submitted exit survey results was different from those who did not. Although the mean cigarettes per day statistic did appear slightly lower, this did not reach statistical significance (20.9 cigarettes per day vs 16.6 cigarettes per day, respectively; *P=*.10).

#### General Responses

Of the exit survey questions that were asked to both groups and where both surveys were completed by the user, there were no significant differences between the groups (Table 3).

**Table 3:** Comparison of the responses to the exit survey questions by both groups.

Response		Control group (n=27), n (%)	Intervention group (n=17), n (%)	*χ* ^2^ *(df)*	*P* value
**The statistics on the Dashboard helped me in my quit attempt**				0.99 (4)	.91
	Strongly disagree	1 (4)	1 (6)		
	Disagree	1 (4)	0 (0)		
	Neutral	6 (22)	4 (24)		
	Agree	11 (41)	8 (47)		
	Strongly agree	8 (30)	4 (24)		
**The educational content in the Information Toolbox helped me in my quit attempt**				1.76 (4)	.78
	Strongly disagree	1 (4)	0 (0)		
	Disagree	1 (4)	0 (0)		
	Neutral	8 (30)	7 (41)		
	Agree	11 (41)	6 (35)		
	Strongly agree	6 (22)	4 (24)		
**The games helped me in my quit attempt**				3.77 (4)	.44
	Strongly disagree	2 (7)	1 (6)		
	Disagree	2 (7)	1 (6)		
	Neutral	14 (52)	9 (53)		
	Agree	5 (19)	6 (35)		
	Strongly agree	4 (15)	0 (0)		
**The games helped me avoid cravings**				4.36 (4)	.36
	Strongly disagree	3 (11)	1 (6)		
	Disagree	1 (4)	2 (12)		
	Neutral	16 (59)	9 (53)		
	Agree	4 (15)	5 (29)		
	Strongly agree	3 (11)	0 (0)		
**How often were you truthful when reporting your daily cigarette intake?**				3.6 (3)	.31
	Never	0 (0)	0 (0)		
	Rarely	1 (4)	1 (6)		
	Sometimes	4 (15)	0 (0)		
	Very often	2 (7)	3 (18)		
	Always	20 (74)	13 (77)		

#### Participant Perceptions of the Games-Based Features of Quittr

The intervention group was asked several questions to ascertain their perceived responses to the games-based features, as shown in Table 4. This group generally showed enthusiasm toward the games features, with the majority of users agreeing that the Tappy Town game was engaging, QuitCoins rewards were enjoyable, and they wanted to earn QuitCoins and spend them in Tappy Town.

**Table 4:** Participants’ perceptions of the games-based features of Quittr (n=17).

Perceptions, responses		n (%), Value
**I found the Tappy Town game engaging**		
	Strongly disagree	1 (6)
	Disagree	0 (0)
	Neutral	6 (35)
	Agree	9 (53)
	Strongly agree	1 (6)
**I enjoyed earning QuitCoins rewards**		
	Strongly disagree	1 (6)
	Disagree	1 (6)
	Neutral	2 (12)
	Agree	11 (65)
	Strongly agree	2 (12)
**I wanted to earn QuitCoins rewards so I could use them in Tappy Town**		
	Strongly disagree	1 (6)
	Disagree	2 (12)
	Neutral	5 (29)
	Agree	8 (47)
	Strongly agree	1 (6)

### Smoking Statistics

Amongst those participants who completed the exit survey, users reported a median daily cigarette reduction of 7 (IQR 0-15) and a weekly median cigarette cost (reported in participant’s own local currency, USD or AUD) reduction of $0 (IQR $0-$19.5). When we compared the groups, these statistics *favored* the control group. The median reduction in daily cigarettes was 12 (IQR 8.5-20) for the control group and 0 (IQR 0-4.5) for the intervention group (*P<*.001), while the median weekly cigarette cost reduction was $8.50 (IQR $0-$46.25) for the control group and $0 (IQR $13-$15) for the intervention group (*P=*.069).

## Discussion

### Principal Findings

Our games-based innovations did successfully increase participant engagement. However, at least with our implementation, their additional engagement was limited to the game itself with little of the desired flow-on effect into other aspects of the app. Nevertheless, it is somewhat encouraging that significant play time was invested into the Tappy Town game, since one of the key concerns with this study was that we would not be able to create a compelling game experience with the time and budget we had available. Unfortunately, the results show that our efforts to incentivize the participants to engage with the broader app were not effective. Similarly, our strategies were not effective in improving the retention of users. However, it is not yet clear whether these failings were due to fundamental flaws with the approach or flaws with our chosen design and implementation.

These relatively poor outcomes become perplexing when we examine the user perceptions from the exit survey, which showed that the majority of users in the game group valued the games features we added and felt that they were incentivized by them. Given this unexpected outcome and the benefit of hindsight, it would have been useful to include survey questions to explore user-perceived engagement in the broader Quittr app, so as to provide insight into whether the user *feels* (dis)engaged by a given aspect of the app and why and to allow more detailed comparisons of engagement between the groups. Nevertheless, the results of this survey combined with the significant number of play-minutes recorded in Tappy Town suggest that our hypothesis may have been undermined by other factors; therefore, significant effort was expended in an attempt to understand what these may be.

### Learnings

Perhaps the most surprising result of the exit survey was that 27 participants in the control group submitted results compared to only 17 in the intervention group and that among these users, the control group achieved significantly better daily cigarette reductions. In effect, what we observed here was that the intervention group had noticeably *worse* retention—the users were less likely to receive or act upon the notification to complete the 28-day exit survey. This prompted significant exploration and discussion among the project team, especially when considered alongside the smoking statistics, which unexpectedly favored the control group.

Considering that the intervention group had access to all the same features and support as the control group, we must consider how this might have occurred. One possible explanation is that the Tappy Town game actively stole users’ attention away from the smoking cessation features of the Quittr app and interfered with the success of their quit attempt. However, users in the intervention group spent just as much time engaging with the smoking cessation content of the app as the control group did; so, this explanation seems unlikely. Another possibility is that the poorer outcome was simply due to chance—this is quite possible, since our sample size was relatively modest and the only statistically significant difference was in the very specific daily cigarette reduction outcome—this could simply be a type I statistical error.

Yet another possibility is that when users grew tired of either the Tappy Town game or the smoking cessation content, they may have uninstalled the entire Quittr app. In contrast, participants in the control group could not become tired of the Tappy Town game; thus, they were less likely to uninstall—this explanation aligns to some degree with activity engagement theory, where experiments have demonstrated that students being given 2 unrelated tasks have their subsequent engagement diminished for both tasks [12].

If we explore theories regarding user retention and engagement through a technology acceptance lens, some other possible explanations arise. It is possible that the addition of the Tappy Town game made the Quittr app feel *less easy to use,* or in other words, required *higher effort* [13,14], or perhaps even made the educational content in the app seem *less credible* [14-16] via its association with a relatively nonserious game experience. Additional research would be required to determine from participants which, if any, of these factors may have come into play and potentially explore strategies to ameliorate the effect. Another likely possibility is that we failed to adequately capture one of the key motivational messages of the SmokeFree-28 app, which quite intensively conveyed the goal of going 28 days smoke-free as part of its PRIME motivational theory-based design. As the authors stated, “the core of SmokeFree-28 involves setting a highly salient target of becoming 28 days smoke-free and monitoring progress towards that target using the app” [4]. Although Quittr did set an equivalent target, it was not as extensively highlighted and focused as in SmokeFree-28; rather, it was just one of many metrics reported on the dashboard—one of the many achievements that could be attained. This may at least partially explain why neither group in our trial managed to achieve equivalent outcomes to the SmokeFree-28 trial (18.9% of the SmokeFree-28 participants successfully reported 28 days of smoke-free status compared to Quittr’s control group at 6%).

Analysis of the SmokeFree-28 trial compared quit success against the Smoking Toolkit Study as a baseline, where 15% of the smokers without any app support were able to successfully quit for 28 days. This baseline still contrasts starkly with our figures, which suggests there may also be fundamental differences in the participants that our respective trials were able to recruit. Perhaps the most logical explanation for this is that the Quittr study was advertised to smokers via social media and targeted web-based advertising, whereas the SmokeFree-28 study appears to have achieved organic participation through user-initiated search and download—this may have created selection bias, with their participants being more self-motivated to quit, whereas our participants were perhaps biased toward strongly identifying as a smoker in their social media presence, and thus becoming the recipient of targeted advertising. This theory is supported when examining our participant profile and recognizing that our participants smoked an average of 20 cigarettes per day and overwhelmingly smoked their first daily cigarette within 30 minutes of waking—this contrasts with the SmokeFree-28 data where ~65% of the participants smoked fewer than 19 cigarettes per day. It appears that our sample was biased toward heavy smokers, which may have made it harder to achieve the 28-day cessation outcome.

There is a final possibility to consider. The exit survey was prompted by a phone notification that occurred after a period of participant inactivity or after the 28-day study period was complete. This feature worked as intended; however, it could be thwarted by users if they explicitly turned off notifications for the Quittr app or uninstalled the Quittr app. It is possible that participants in the intervention group may have been more likely to turn off notifications or uninstall the app. The app would send notifications once daily at 8 PM. There were 3 reminders, which would fire if appropriate: one to remind the user to log their cigarette usage, which would always fire, one to prompt them to complete the exit survey if the user had been inactive for an extended period or had reached 28 days of participation, and a third that only applied to intervention group users, which would prompt the user to spend excess resources in Tappy Town if their accrued resources were over a given threshold. Consequently, most participants in the intervention group would receive 2 simultaneous notifications each night, whereas most control participants would receive only 1. We suspect this “spammy” activity may have prompted some of them to turn off the notifications or uninstall the app.

Unfortunately, we have no way of knowing whether they did turn off notifications or uninstall the app, or when. However, it may also explain why we saw no positive intervention effect on retention in general—if participants in the intervention group were more likely to turn off notifications, then they were missing out on a critical aspect of our user retention strategy: daily reminders to log in, log cigarette usage, and generally engage with the content of the app. In light of this, it is appropriate that future studies should examine their data collection strategies with care and ideally include features to log whether notifications for the app were disabled and attempt to track when the app is uninstalled.

There is another result that suggests there may be potential for design improvement. Only 9 of the 17 respondents (53%) reported wanting to earn QuitCoins to spend them in Tappy Town compared to 13 (77%) who said they enjoyed earning QuitCoins. This difference may simply be that some users were not motivated by the Tappy Town game but did like the feeling of achievement that the QuitCoins rewards gave them. However, on reflection, we suspect that more should have been done to make the link between earning QuitCoins and spending them in Tappy Town. There were no explicit messages or reminders for the user to earn and spend QuitCoins, and at very few points, it was explained to the user that QuitCoins could be spent in Tappy Town to build powerful structures. This is unlike most mobile games that use a similar premium currency model.

### Conclusions and Further Work

Although our pilot study achieved poor results in terms of both the primary and secondary outcomes, it has nevertheless provided some useful insights and some potentially encouraging preliminary data. Survey responses suggest that the hypothesis “games features can be used to encourage engagement and retention in a quit smoking app” has some credibility on face value at least, and the significant play-time invested by participants into the Tappy Town game suggests that the game itself was acceptable. It appears as if there may be other factors at play that adversely affected the outcomes of the intervention group, although perhaps the game itself negatively affected retention through one of the discussed theoretical mechanisms or a mechanism still unknown.

Without these adverse factors in play, would a positive outcome have been achieved? To answer that question, it would be necessary to categorically identify and fix the issues before rerunning the experiment. Although we have identified some potential issues, this study was not designed for this purpose; therefore, there may be other issues yet unidentified. We would recommend commissioning first a smaller scale usability analysis with observed and qualitatively studied users. This study should identify potential pain points and points of conflict that could negatively impact the outcomes of the intervention, which can then be remedied before undertaking any further quantitative study.

## Appendix

Multimedia Appendix 1 CONSORT-EHEALTH checklist (V 1.6.1).

Multimedia Appendix 2 Media release and Twitter advertisements.

Multimedia Appendix 3 Entry and exit surveys.

## Abbreviations

mHealth: mobile health
