# Supplementary material for: Effects of a Mobile App Called Quittr, Which Utilizes Premium Currency and Games Features, on Improving Engagement With Smoking Cessation Intervention: Pilot Randomized Controlled Trial
Source: JMIR Serious Games. 2020 Dec 14;8(4):e23734. doi: 10.2196/23734 (PMC7769690; doi:10.2196/23734)

## Appendix A

The University of Tasmania, with the support of the Heart Foundation of Australia Vanguard Grants program, is proud to release an innovative new smoking cessation app on the Google Play and iTunes stores.

Quittr aims to be the fun way to quit smoking. It includes a wealth of behavioural support content, personalised statistics, an achievements system, and enjoyable games that reward the smoker for persisting and engaging with their quit attempt.

The Quittr app is completely free and is available now in the Google Play and iTunes stores. Users will be enrolled in a pilot study, providing valuable data to determine how effective the app is at helping smokers to quit.

“We know that smoking cessation apps can be effective, but it’s really hard to keep smokers engaged beyond the first few days. Quittr makes use of techniques from the games industry to try and improve in this area.” Dr Ivan Bindoff, Chief Investigator. “Our goal was to make the process feel genuinely rewarding, right from the start.”

To find out more and download the app visit <https://www.pharm.utas.edu.au/scg/>

**Please feel free to share amongst appropriate networks and social media.**

*Suggested Tweets – suitable partner images included below:*

Can quitting smoking really be fun? The #Quittr app thinks so. @UTAS_ @vanmani <https://www.pharm.utas.edu.au/scg/>

Do games have a role to play in effective smoking cessation? #Quittr study from @vanmani @UTAS_ aims to find out. <https://www.pharm.utas.edu.au/scg/>

Interested in quitting smoking? Enrol in the Quittr study and get an app to help with that. #Quittr @vanmani @UTAS_ <https://www.pharm.utas.edu.au/scg/>


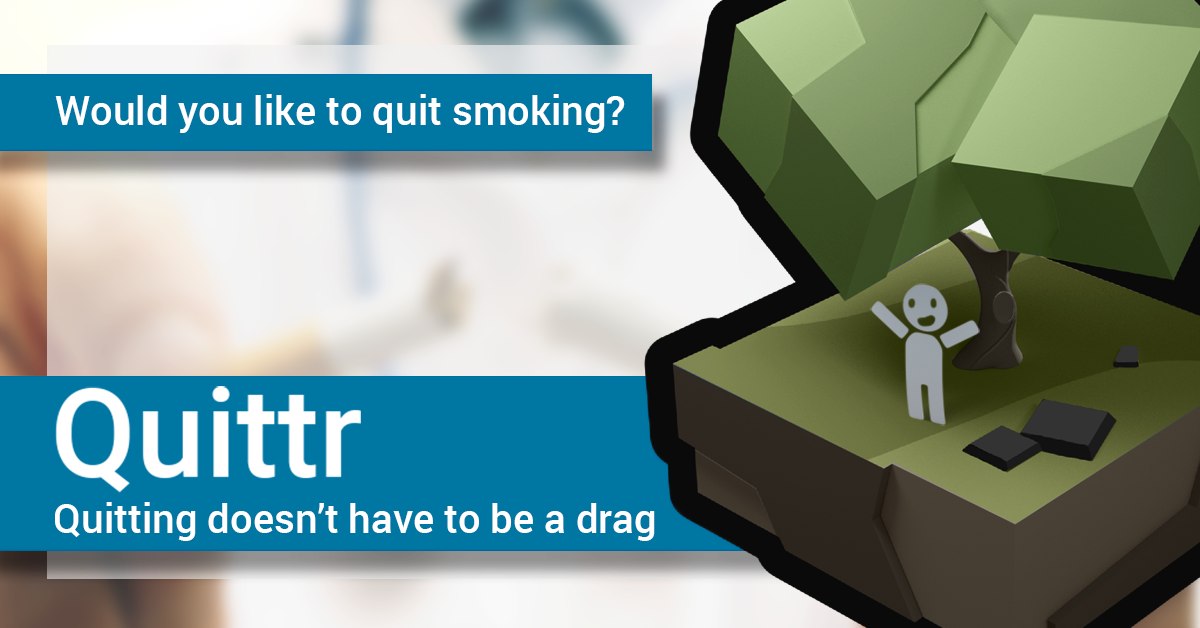

Supplement: Multimedia Appendix 2 [file games_v8i4e23734_app2.docx]
