# Supplementary material for: Effects of a Mobile App Called Quittr, Which Utilizes Premium Currency and Games Features, on Improving Engagement With Smoking Cessation Intervention: Pilot Randomized Controlled Trial
Source: JMIR Serious Games. 2020 Dec 14;8(4):e23734. doi: 10.2196/23734 (PMC7769690; doi:10.2196/23734)
Supplement: Multimedia Appendix 3 [file games_v8i4e23734_app3.docx]

## Appendix B

Entry survey – triggers when user first opens the Quittr app

1. Are you currently a smoker? [Yes/No] (If No, skip to Q5)
2. How much do you spend on cigarettes per week? [Dollars]
3. How many cigarettes do you smoke per day on average? [Integer]
4. How soon after waking do you smoke your first cigarette? [Within 5 minutes/6-15 Minutes/16-30 Minutes/31 Minutes to an hour/More than 1 hour]
5. Why are you installing Quittr? [Just testing/To play games/<To quit smoking – this only applies if indicated Yes in Q1>]
6. How old are you? [Years]
7. What is your sex? [Male/Female]
8. [If Female] Are you currently pregnant? [Yes/No]
9. Are you intending to use Nicotine Replacement Therapy during your quit attempt? (i.e. patch, gum, inhaler, etc.) [Yes/No]
10. [If yes] What form of Nicotine Replacement Therapy will you use? [Patch/Gum|Lozenge|Inhaler|Spray/Combination]
11. Are you intending to use a medication to reduce cravings/withdrawals? [Yes/No]
12. [If yes] What form of medication will you use? [Varenicline (i.e. Champix, Chantix)/Bupropion (i.e. Wellbutrin, Zyban)/Both]

Exit survey – triggers after 28 days, or when the user indicates their quit attempt is over. Only applies to users who indicated Yes in Q1.

1. How much do you currently spend on cigarettes per week? [Dollars]
2. How many cigarettes do you smoke per day on average? [Integer]
3. I found the Tappy Town game engaging. [Likert - SA/A/N/D/SD]
4. I enjoyed earning QuitCoin rewards. [Likert - SA/A/N/D/SD]
5. I wanted to earn QuitCoin rewards so I could use them in Tappy Town. [Likert - SA/A/N/D/SD]
6. The statistics on the Dashboard helped me in my quit attempt. [Likert - SA/A/N/D/SD]
7. The educational content in the Information Toolbox helped me in my quit attempt. [Likert - SA/A/N/D/SD]
8. The games helped me in my quit attempt. [Likert - SA/A/N/D/SD]
9. The games helped me avoid cravings. [Likert - SA/A/N/D/SD]
10. How often were you truthful when reporting your daily cigarette intake? [Likert – Always/Very Often/Sometimes/Rarely/Never]
